# Unilateral Choanal Atresia in a Child With Prolonged Nasal Congestion

**DOI:** 10.7759/cureus.57669

**Published:** 2024-04-05

**Authors:** Lindsay Ussher, Carli David, Randall Hansen, Alex Otto, Scott McClintick, Kent McIntire, Suporn Sukpraprut-Braaten

**Affiliations:** 1 Surgery, Kansas City University of Medicine and Biosciences, Joplin, USA; 2 Surgery, Edward Via College of Osteopathic Medicine, Monroe, USA; 3 Otolaryngology-Head and Neck Surgery, Freeman Health System, Joplin, USA; 4 Otolaryngology-Head and Neck Surgery, Graduate Medical Education, Kansas City University, Joplin, USA; 5 Biostatistics, Epidemiology, and Public Health, Graduate Medical Education, Kansas City University, Kansas City, USA

**Keywords:** inferior turbinate hypertrophy, nasal congestion, diagnosis, choanal atresia, right unilateral

## Abstract

Choanal atresia obstructs the nasal passage due to abnormal bony or soft tissue remnants owing to the faulty canalization of the nasal passages during fetal development. The clinical manifestations are more pronounced in bilateral cases, often presenting immediately after birth with cyanosis turning pink when crying, as newborns are obligatory nasal breathers. This contrasts in unilateral cases, where the condition may present with mild symptoms and be diagnosed later in life. We present a case of a five-year-old male who initially presented with a concern for nasal polyps due to nasal congestion with absent airflow out of the right nostril. On examination of the pharynx and nose, the patient was diagnosed with nasal turbinate hypertrophy, the right more than the left, and was subsequently scheduled for bilateral inferior turbinate reduction, possible adenoidectomy, and nasal endoscopy. Intraoperatively, inspection with nasal endoscopy along with the inability to pass a catheter through the nasopharynx to reach the oropharynx was our indicator of a more severe diagnosis. Here, we report an incidental finding of the right choanal atresia and seek to highlight its importance given this incidental finding.

## Introduction

Choanal atresia is an obliteration of the posterior choanae of the nasal cavity [1]. It arises from the medialization of the lateral pterygoid plate (resulting in the lateral obstruction) and an enlarged vomer (resulting in the medial aspect of the obstruction) [2]. Choanal atresia was first described in 1755 by Johann Roderer during the evaluation of a newborn, and in 1854, Emmert reported the first successful operation for treating congenital choanal atresia via the transnasal approach [3]. Bilateral cases may be life-threatening, with symptoms of upper airway obstruction almost always prompting early detection in infants and neonates [4]. Alternatively, unilateral cases may go undetected for years, with more subtle cases being detected in adulthood [5]. Such cases can prove perplexing for physicians since a high index of suspicion is required to make the diagnosis. An example of a masquerading diagnosis is demonstrated by Rothman et al. who reported a case of a nine-year-old male who presented with rhinorrhea, chronic mouth breathing, and occasional snoring. He was evaluated by a pediatrician who noted the improvement of symptoms after antibiotics. Plain sinus radiographs in conjunction with multiple courses of antibiotics were interpreted as "sinusitis." He was later referred to a pediatric allergist where a CT scan demonstrated chronic ethmoid mucoperiosteal thickening. He was subsequently referred to an otolaryngologist who later correctly identified the underlying cause [6]. Additionally, Joshua et al. reported five cases of unilateral choanal atresia. While two adults had undergone unnecessary septal and turbinate surgery, two older children were treated medically, and one was treated for epiphora [7]. Kilic et al. reported an incidental finding of left unilateral choanal atresia in a 25-year-old female only observed during an endoscopic examination after the surgery correction of severe septal deviation [8]. Finally, Coggins and McDonald reported that a 34-year-old female post COVID-19 infection who presented with symptoms of anosmia, ageusia, and headache was diagnosed with inferior turbinate hypertrophy and deviated nasal septum. She underwent inferior turbinate reduction and nasal septoplasty with minimal improvement. Upon revaluation, left unilateral choanal atresia was appreciated, and subsequent surgical repair resolved her symptoms [9].

Attention is given to presenting these examples in chronological order spanning a period of 28 years to highlight the persistence of misdiagnosis often due to atypical presentation coupled with a low index of suspicion. We anticipate that cases of a similar nature have occurred within this period; therefore, such cases highlight the continued difficulty in elucidating the accurate diagnosis particularly when symptoms are masked by more frequent pathologies. We report a case of a five-year-old child who presented with symptoms of nasal congestion and absent airflow from the right nostril.

## Case presentation

This case report details the clinical presentation of a previously healthy five-year-old male who presented to the otolaryngology outpatient department with a primary complaint of nasal congestion. His mother reported that the child had been using nasal steroids intermittently for allergies and nasal congestion. The mother denied any observable masses, recurrent episodes of upper respiratory infections, or nasal discharge. A comprehensive symptom review was conducted, yielding negative results, including an absence of dysphagia, choking episodes, fever, or headaches. The patient's family history was significant for diabetes, but his social history was otherwise unremarkable and noncontributory. The initial anterior rhinoscopy of the nostrils with a light source revealed the bilateral hypertrophy of the inferior nasal turbinates. The remaining examination of the patient's neck, pharynx, nose, and ears appeared normal. The diagnosis of nasal turbinate hypertrophy was made, predominantly on the right side.

**Figure 1:**
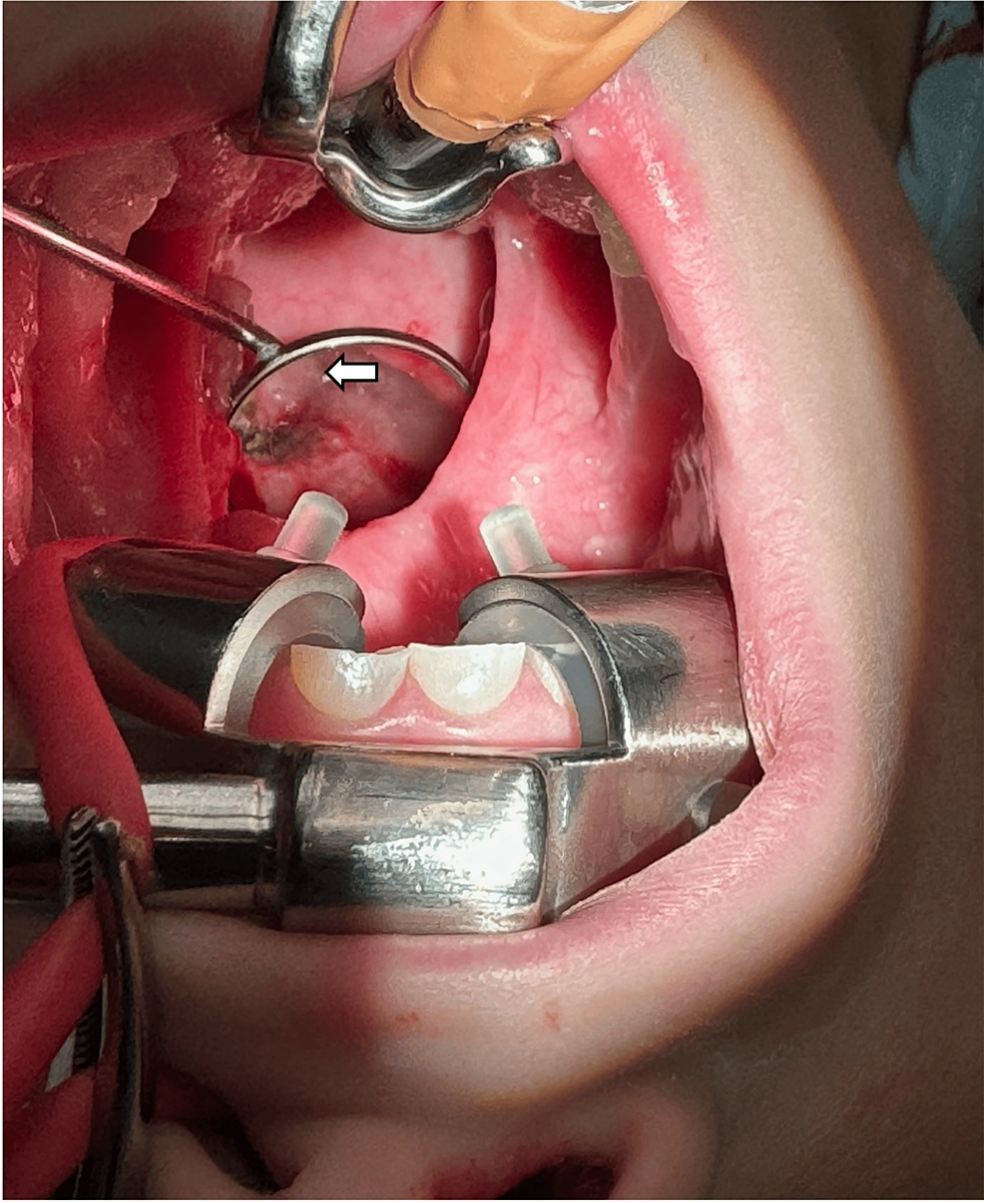
Posterior rhinoscopy visualizing the right unilateral choanal atresia (white arrow)

Due to the patient's lack of cooperation, we did not pursue nasal endoscopy with further imaging at the time of presentation in the clinic. Instead, the decision was made to schedule inferior turbinate reduction with possible adenoidectomy and nasal endoscopy. This approach would address the identified obstructive pathology and rule out other potential causes of obstruction simultaneously. At the time of the planned procedures, attention was first brought to nasal endoscopy, which allowed us to identify the unilateral choanal atresia. Next, the submucosal bilateral inferior turbinate reduction was performed. Injectable saline was utilized on both turbinates, and a Celon device was positioned and held in place for approximately 15 seconds on both sides. Once the turbinates were successfully outfractured, attention was turned to performing the adenoidectomy. A red rubber catheter was utilized and could not be passed through the right nasal cavity; however, it could successfully pass through the left side. After fulgurating the obstructive adenoid tissue in a standard midline fashion, the right choana was further inspected with the utilization of a dental mirror and posterior rhinoscopy, which demonstrated a membranous wall consistent with choanal atresia on the right side, confirming the diagnosis (Figure 1). The surgery to correct the patient's defect was not undertaken due to its higher level of complexity. We determined that it was appropriate to refer the patient to a tertiary facility capable of providing a suitable treatment protocol including necessary imaging such as CT scans for preoperative planning, with a likely procedure being transnasal endoscopic choanoplasty. There were no complications, and the patient was extubated and referred to a children's hospital for further management.

## Discussion

Choanal atresia is a less common cause of nasal obstruction, and controversy remains over the exact pathogenesis since its first description in the early 18th century [10]. Currently, multiple theories regarding the developmental process are accepted: the persistence of the bucconasal membrane, aberrant neural crest cell migration into the nasal vault, and abnormal persistence of mesoderm leading to the formation of adhesions of the nasochoanal region [10,11]. Such pathophysiological defects may result in a complete bony (30%), mixed bony-membranous (70%), or membranous (rare) defect of the posterior nasal cavity, with pterygoid plate and vomer forming its components [11].

Some older studies suggest single-gene models that include autosomal dominant and recessive transmission [12], while others suggest nongenetic factors contributing in a multifactorial manner [13]. Still, many studies have demonstrated choanal atresia's association with common phenotypes known as the coloboma, heart defect, atresia of choanae, retarded growth and development, genital anomaly, and ear defects (CHARGE) syndrome. The resulting mutations in the *CDH7* gene are found, yet the CHARGE syndrome is associated with approximately 50% of cases of choanal atresia [14]. It could be argued that in such a context, additional phenotypes may prompt further examination and increase the likelihood of diagnosis. Cases presenting as described may pose more significant challenges in determining the cause of symptoms [15], as demonstrated in a similar case of a five-year-old female misdiagnosed with adenoid hypertrophy. Right choanal obstruction was later appreciated during surgery [16]. We present this case report to further stress the difficulties in accurately diagnosing such presentations when additional phenotypic traits are absent.

Conversely, we briefly highlight undiagnosed cases of unilateral choanal atresia, holding equal significance in this discussion. Mohammadi reported 11 patients within the age range of 11-15 who presented with symptoms of unilateral nasal discharge, snoring, and nasal blockage. They were found to have undiagnosed unilateral choanal atresia and underwent subsequent surgical repair with no complications [17]. In another study, Ferraria et al. reported a 48-year-old female who presented with a lifelong history of tiredness. Incidental findings of clear profuse right nostril mucoid discharge and obstruction prompted the suspicion for unilateral choanal atresia [18]. Similarly, another study documents four distinct cases of unilateral choanal atresia diagnosed in adulthood [19]. Considering that such studies highlight the delay in diagnosis, we emphasize that they represent genuine undiagnosed cases, distinguishing them from those typically diagnosed later in life due to less severe symptoms [20,21]. Collectively, we seek to demonstrate the importance of both misdiagnosed and undiagnosed cases, underscoring how they reflect often variable and ambiguous symptomatology. Bilateral choanal atresia is far less likely to be misdiagnosed due to its highly conspicuous features of respiratory distress immediately after birth [22,23]. Its counterpart, unilateral choanal atresia, often presents with unilateral nasal congestion and mucopurulent discharge [24]. Additionally, the lack of cyanosis, noisy breathing, and periodic respiratory distress may contribute to the frequently delayed diagnosis of unilateral choanal atresia, due to its non-life-threatening symptoms. Yet, the inability to pass the nasogastric tube may quickly raise the suspicion for unilateral cases in non-acute patients (Table 1). Possible differential diagnoses include antrochoanal polyp, chordoma, deviated nasal septum, dislocated nasal septum, hematoma, isolated pyriform aperture stenosis, nasal dermoid, nasolacrimal duct cyst, septal dislocation, and turbinate hypertrophy [10,25-28].

**Table 1:** Comparison of symptoms between unilateral choana, atresia, and bilateral choanal atresia Source: [23]

Symptoms	Unilateral choanal atresia	Bilateral choanal atresia
Persistent nasal drainage	Yes	No
Feeding difficulties	Yes	Yes
Medical emergency in neonatal period	No	Yes
Noisy breathing	No	Yes
Mouth breathing	Yes	No
Cyclic respiratory distress	No	Yes
Cyanosis	No	Yes
Inability to pass nasogastric tube into the nasopharynx	Yes	Yes

The diagnosis of unilateral choanal atresia is easily distinguishable with CT [29]. Yet, most cases may go undiagnosed until later in life, particularly in teens, especially if there is no high index of suspicion in patients frequently presenting to the clinic due to persistent unilateral nasal obstruction [22]. Since CT scans are not used as routine for chronic nasal obstruction, a large proportion of cases such as these described result in a delayed diagnosis. As noted earlier, we discuss an unusual case related to the events that preceded surgery. Since nasal endoscopy was not feasible due to the patient's intolerance, we performed nasal endoscopy alongside other planned procedures at the time of surgery. Typically, nasal endoscopy would prompt further workup with imaging prior to the operation. Interestingly, Tatar et al. also reported alternative methods of diagnosing the condition without imaging, such as using a nasopharyngeal mirror or finger examination [30]. Still, such methods likely yield considerably lower sensitivity than imaging. With cases ranging from 1:5000 to 1:8000 births, we speculate that many practitioners may view such methods with utility rather than opting to pursue imaging should their suspicion arise. This, coupled with subtle symptoms of unilateral choanal atresia, may contribute heavily to a delayed diagnosis.

The surgical management of choanal atresia consists of a variety of approaches. The most preferred is the endoscopic transnasal approach due to the adequate exposure of the choanal plate and minimizing the risk of restenosis. Other techniques include transnasal or transpalatal resection [22].

## Conclusions

Unilateral choanal atresia can present in childhood with symptomatology mimicking more commonly seen obstructive causes. A high index of suspicion by clinicians is necessary for establishing the diagnosis of unilateral choanal atresia; otherwise, it may be missed until adulthood. Physicians should consider the diagnosis of unilateral choanal atresia as a possibility in children presenting with obstructive pathology. Alternative methods including a finger examination and nasopharyngeal mirror may be tried to elucidate the cause prior to definitive imaging. Primary care physicians may serve as the lead point in the initial suspicion of choanal atresia, with referral to an otolaryngologist being critical in obtaining a precise diagnosis. Transnasal endoscopic choanoplasty is the most preferred technique for surgical repair.

## Figures and Tables

**Figure 2:**
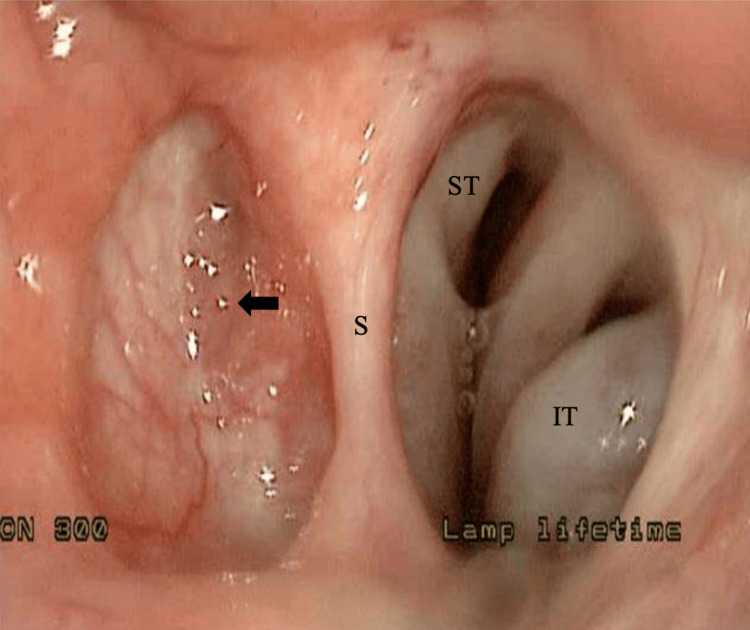
Retropalatal endoscopic view of the membranous unilateral choanal atresia (black arrow) Source: [31] IT, interior turbinate; S, septum; ST, superior turbinate

## Appendices

We show a retropalatal endoscopic view of the right unilateral choanal atresia (Figure 2) to demonstrate its often mixed nature of membranous and bony elements.
